# Microbial (*E*)-4-hydroxy-3-methylbut-2-enyl pyrophosphate reductase (IspH) and its biotechnological potential: A mini review

**DOI:** 10.3389/fbioe.2022.1057938

**Published:** 2022-11-29

**Authors:** Shiyong Huang, Yanfen Xue, Yanhe Ma, Cheng Zhou

**Affiliations:** ^1^ State Key Laboratory of Microbial Resources, Institute of Microbiology, Chinese Academy of Sciences, Beijing, China; ^2^ University of Chinese Academy of Sciences, Beijing, China

**Keywords:** IspH, MEP pathway, HMBPP reductase, structural properties, catalytic mechanism, biotechnological potential

## Abstract

(*E*)-4-hydroxy-3-methylbut-2-enyl pyrophosphate (HMBPP) reductase (IspH) is a [4Fe-4S] cluster-containing enzyme, involved in isoprenoid biosynthesis as the final enzyme of the methylerythritol phosphate (MEP) pathway found in many bacteria and malaria parasites. In recent years, many studies have revealed that isoprenoid compounds are an alternative to petroleum-derived fuels. Thus, ecofriendly methods harnessing the methylerythritol phosphate pathway in microbes to synthesize isoprenoid compounds and IspH itself have received notable attention from researchers. In addition to its applications in the field of biosynthesis, IspH is considered to be an attractive drug target for infectious diseases such as malaria and *tuberculosis* due to its survivability in most pathogenic bacterium and its absence in humans. In this mini-review, we summarize previous reports that have systematically illuminated the fundamental and structural properties, substrate binding and catalysis, proposed catalytic mechanism, and novel catalytic activities of IspH. Potential bioengineering and biotechnological applications of IspH are also discussed.

## Introduction

Isoprenoids, including steroids and terpenes, are one of the largest and most diverse classes of natural products. They include essential biological compounds such as vitamins, cholesterol, steroid hormones, carotenoids, and quinines (Rohmer, 1999; Eisenreich et al., 2004). In organisms they are derived from the same precursors: isopentenyl diphosphate (IPP) and dimethylallyl diphosphate (DMAPP) (Oldfield and Lin, 2012). Two distinct biosynthetic pathways are known to produce both IPP and DMAPP: the mevalonate (MVA) pathway, which is present in mammals as well as some microorganisms, and the methylerythritol phosphate (MEP) pathway, found in many pathogenic bacteria such as *Mycobacterium tuberculosi* and *Plasmodium falciparum* (Kuzuyama and Seto, 2003).

**FIGURE 1:**
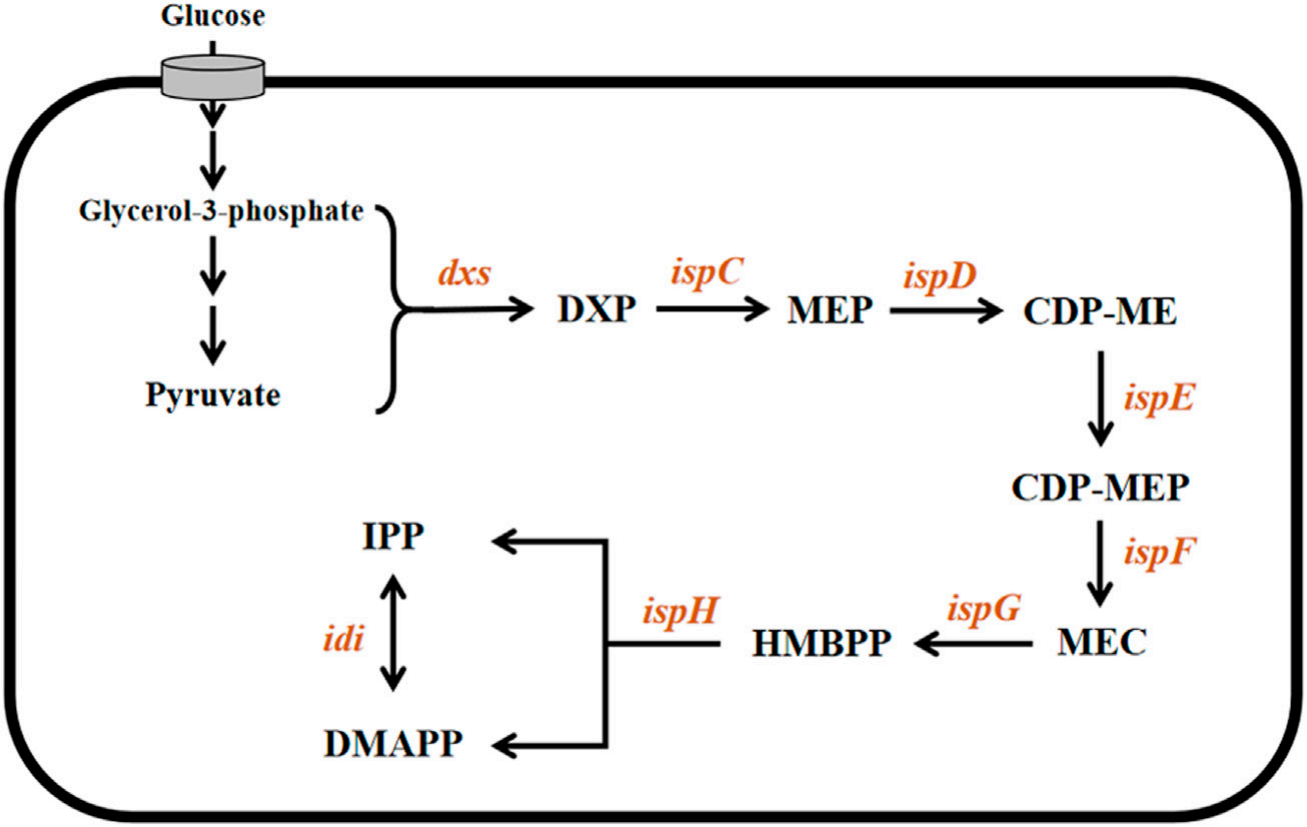
Microbial MEP pathway. The involved genes and corresponding enzymes are *dxs*: 1-deoxyxylose-5-phosphate synthase; *ispC*: 1-deoxyxylose-5-phosphate reductoisomerase; *ispD*: 2-C-methyl-D-erythritol-4-phosphate cytidyltransferase; *ispE*: 4-(cytidine-5′-diphosphate)-2-c-methyl-d-erythritol kinase; *ispF*: 2-C-methyl-D-erythritol-2,4-cyclodiphosphate synthase; *ispG*: 4-hydroxy-3-methyl-2-(*E*)-butenyl pyrophosphate synthase; and *ispH* (*E*)-4-hydroxy-3-methylbut-2-enyl pyrophosphate reductase. The involved intermediate metabolites are DXP: 1-Deoxy-d-xylulose-5-phosphate; MEP: 2*C-*methyl-D-erythritol-4-phosphate; CDP-ME: 4-(cytidine-5′-diphosphate)- 2-c-methyl-d-erythritol; CDP-MEP: 2-C-methyl-D-erythritol-2,4-cyclodiphosphate; MEC: 4-hydroxy-3-methyl-2-(E)-butenyl pyrophosphate; and HMBPP (*E*)-4-hydroxy-3-methylbut-2-enyl pyrophosphate.

The MEP pathway (Figure 1) begins with the condensation of pyruvate and glyceraldehyde 3-phosphate to form 1-deoxy-d-xylulose-5-phosphate (DXP), catalyzed by the enzyme 1-deoxyxylulose-5-phosphate synthase (DXS). DXP is then converted into 2-C-methyl-D-erythritol-4-phosphate by IspC and is also called 1-deoxyxylulose-5-phosphate reductoisomerase (DXR). A sequence of steps catalyzed by the enzymes IspD, IspE, and IspF converts 2-C-methyl-D-erythritol-4-phosphate into 2-C-methyl-D-erythritol-2,4-cyclodiphosphate *via* cytidine diphosphate intermediates (4-diphosphocytidyl-2-C-methyl-D-erythritol and 4-diphosphocytidyl-2-C-methyl-D-erythritol-2-phosphate). IspG/GcpE catalyze the penultimate reaction in the pathway by reducing and opening the cyclic diphosphate intermediate to form (*E*)-4-hydroxy-3-methylbut-2-enyl pyrophosphate (HMBPP). The final step is the conversion of HMBPP into a mixture of IPP and DMAPP (Wang et al., 2012; Span et al., 2014). This reductive dehydroxylation (Figure 1) is catalyzed by IspH, an oxygen-sensitive monomeric protein with a [4Fe-4S] cluster at the active site. Because it plays a key role in the biosynthesis of isoprenoids and is essential for survival, IspH has attracted great interest, particularly with regard to the development of new antimicrobial drugs as well as novel biofuels as alternatives to petroleum-derived fuels.

In this mini-review, we summarize the fundamental features of IspH, its structural properties, substrate binding and catalysis, proposed catalytic mechanisms, and novel catalytic activities. Potential bioengineering and biotechnological applications of IspH are also discussed.

### Fundamental features of IspH

As an enzyme in the MEP pathway, IspH occurs in most bacteria, plant chloroplasts, green algae, and apicomplexan, but it is absent in humans (Rohmer et al., 2004). The *lytB* gene, encoding LytB (EC.1.17.7.4), also called IspH, was first described in *Escherichia coli* as a gene involved in penicillin resistance (Gustafson et al., 1993) and was later reported to be present in other bacteria (Potter et al., 1998). Cunningham *et al.* discovered that the deletion of *lytB* from *Synechocystis* PCC6803 was fatal, but the strain was able to recover when supplied with an analog of either IPP or DMAPP (3-methyl-3-butene-1-alcohol or 3-methyl-2-butene-1-alcohol, respectively) in the culture medium. Moreover, *lytB* can increase the biosynthesis of carotenoids when expressed in *E. coli* (Cunningham et al., 2000). These findings confirm that *lytB* is involved in the MEP pathway. Since these studies, a series of *in vitro* experiments have shown that IspH is the last enzyme in the MEP pathway, responsible for the conversion of HMBPP to IPP and DMAPP (Altincicek et al., 2002; Petra et al., 2002).

Because IspH contains an oxygen-sensitive [4Fe-4S] cluster at its active site, it is easily oxidized and inactivated when exposed to air. Thus, purification of this protein is usually carried out in an anaerobic chamber. Gräwert *et al.* reported that a purified (in anaerobic conditions) solution of IspH was iron-green in color. When exposed to air for 1 h, the protein gradually deactivated and turned light brown (Gräwert et al., 2010). Decomposition of the oxygen-sensitive [4Fe-4S] cluster in IspH leads to partial loss of the tertiary structure and to complete loss of function, as also observed for aconitase (Kent et al., 1982) and radical *S*-adenosyl-l-methionine (SAM) enzymes (Tamarit et al., 2000; Layer et al., 2004).

Numerous reports on the biochemical properties and enzyme kinetics of IspH also exist. Altincicek *et al.* determined that the optimum pH of *Aquifex aeolicus* IspH (AaIspH) was in the range 7.0–7.5. Activity was observed at 30°C–70°C, with maximum activity occurring at 60°C. The K_m_ value of AaIspH for HMBPP was 590 ± 60 μM (Altincicek et al., 2002).

### Structure, substrate binding, and catalysis of IspH

**FIGURE 2:**
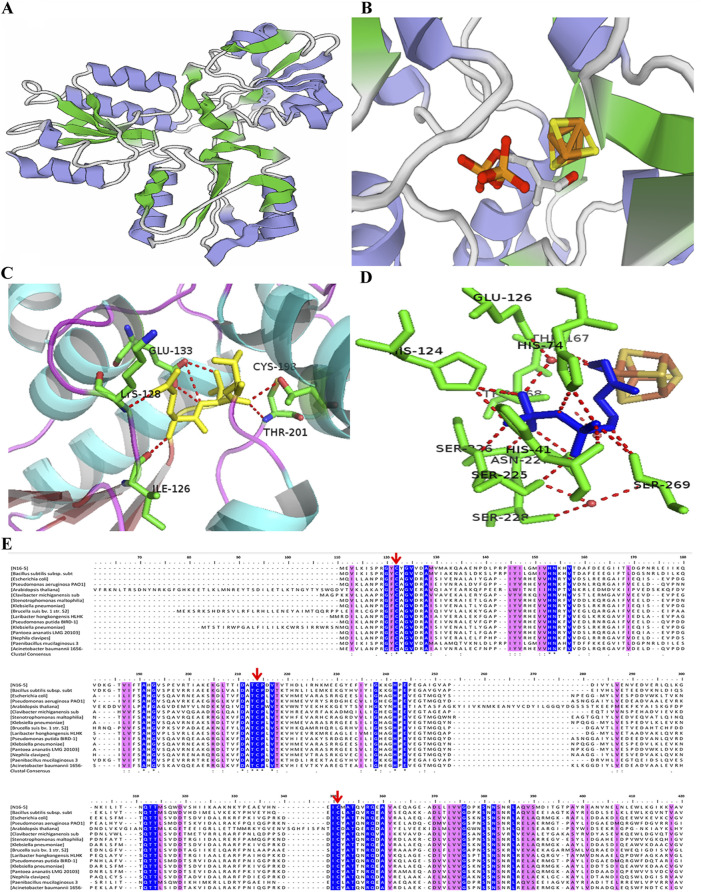
Structure, active sites, and multiple sequence alignment of IspHs from different species. **(A)** Three-dimensional structure model of monomeric IspH. **(B)** Crystal structure of the IspH-HMBPP complex; the [4Fe-4S] cluster center is shown as a ball-and-stick model with iron atoms colored in orange and sulfur atoms in gold. **(C)** Binding of HMBPP at the active site in IspH. HMBPP is shown as a ball-and-stick model in yellow. **(D)** Binding of HMBPP shown as a ball-and-stick model in blue and the hydrogen-bonding network shown by the dotted red lines. **(E)** Multiple sequence alignment of IspHs in different species. Cysteines coordinating the iron sulfur cluster are indicated by the red arrows. Stringently conserved residues are highlighted in blue.

Even though IspHs from different microorganisms exhibit a low sequence homology, for example, the sequence similarity of *E. coli* and *A. aeolicus* IspHs is only 43%, high structural similarity between different IspHs is observed. X-ray crystallographic structures of IspHs are available for the protein in *E. coli* (EcIspH, PDB: 3F7T) (Gräwert et al., 2010), *A. aeolicus* (AaIspH, PDB: 3DNF) (Rekittke et al., 2008), and *P. falciparum* (PfIspH, PDB: 4N7B) (Rekittke et al., 2013). These structures present a similar “trefoil” arrangement consisting of three α/β domains with the Fe-S cluster bound at the center of the structure (Figures 2A, B). The [4Fe-4S] cluster is usually present in the crystallographic structure of IspH. However, the crystal structure of IspH in *A. aeolicus* exhibited a [3Fe-4S] center; it is believed that the [3Fe-4S] center observed in the crystal structure lost an iron atom during co-crystallization with HMBPP (Rekittke et al., 2008). In functional IspHs, the oxidized cluster is in the form [4Fe-4S]^2+^, which is bound to three highly conserved cysteine residues. The fourth (unique) Fe atom is involved in ligand binding and electron transport during catalysis.

The catalytic reaction of IspH is a redox reaction. The ligand can be an electron donator, such as a flavodoxin/flavodoxin reductase/NADH system (*E. coli*) (Wolff et al., 2002; Puan et al., 2005), or ferredoxin (in *P. falciparum*) (Röhrich et al., 2005), and is essential during catalysis. Chemical reductants, such as artificial electron donors including photoactivated deazaflavin (Xiao et al., 2008) and methyl dithionite (Altincicek et al., 2002), can usually be used in *in vitro* experiments (Gräwert et al., 2009).

Co-crystallization of an enzyme with a substrate is the common method to explore the substrate binding and to identify active regions of the enzyme. Although most studies have merely obtained the ligand-free IspH structure which is challenging to co-crystallize with a substrate, the crystal structure of the EcIspH-ligand complex has been obtained and analyzed. In the complex structure, the HMBPP substrate interacted with multiple amino acid residues *via* hydrogen bonding. The substrate-binding region and the key binding sites in IspH were determined (Figures 2C, D) (Rekittke et al., 2013; Xu et al., 2016). A number of amino acids residues were very highly conserved in different IspHs (Figure 2E), and these residues are mainly involved in catalysis or maintaining enzyme activity. Three cysteine residues (Cys12, Cys96, and Cys197 in *E. coli* IspH) bind to the iron-sulfur center; mutation of any one of these residues causes a complete loss of enzymatic activity (Petra et al., 2002; Gräwert et al., 2010). In *A. aeolicus* IspH, three histidine residues, His42, His74, and His124, are also involved in substrate binding (Gräwert et al., 2009). Another conserved amino acid, Glu126 in *E. coli* IspH, has been shown to play a crucial role as a proton donor during catalysis (Span et al., 2012a). The residue His41 in *E. coli* IspH (His42 in *A. aeolicus* IspH) is predicted to be involved in the binding of HMBPP, is essential for catalysis, and may be involved in delivering H^+^ from Glu126 to the bound HMBPP. The residue Thr167 may act as a proton relay and Glu126 serves as the ultimate proton donor (Gräwert et al., 2009).

**FIGURE 3:**
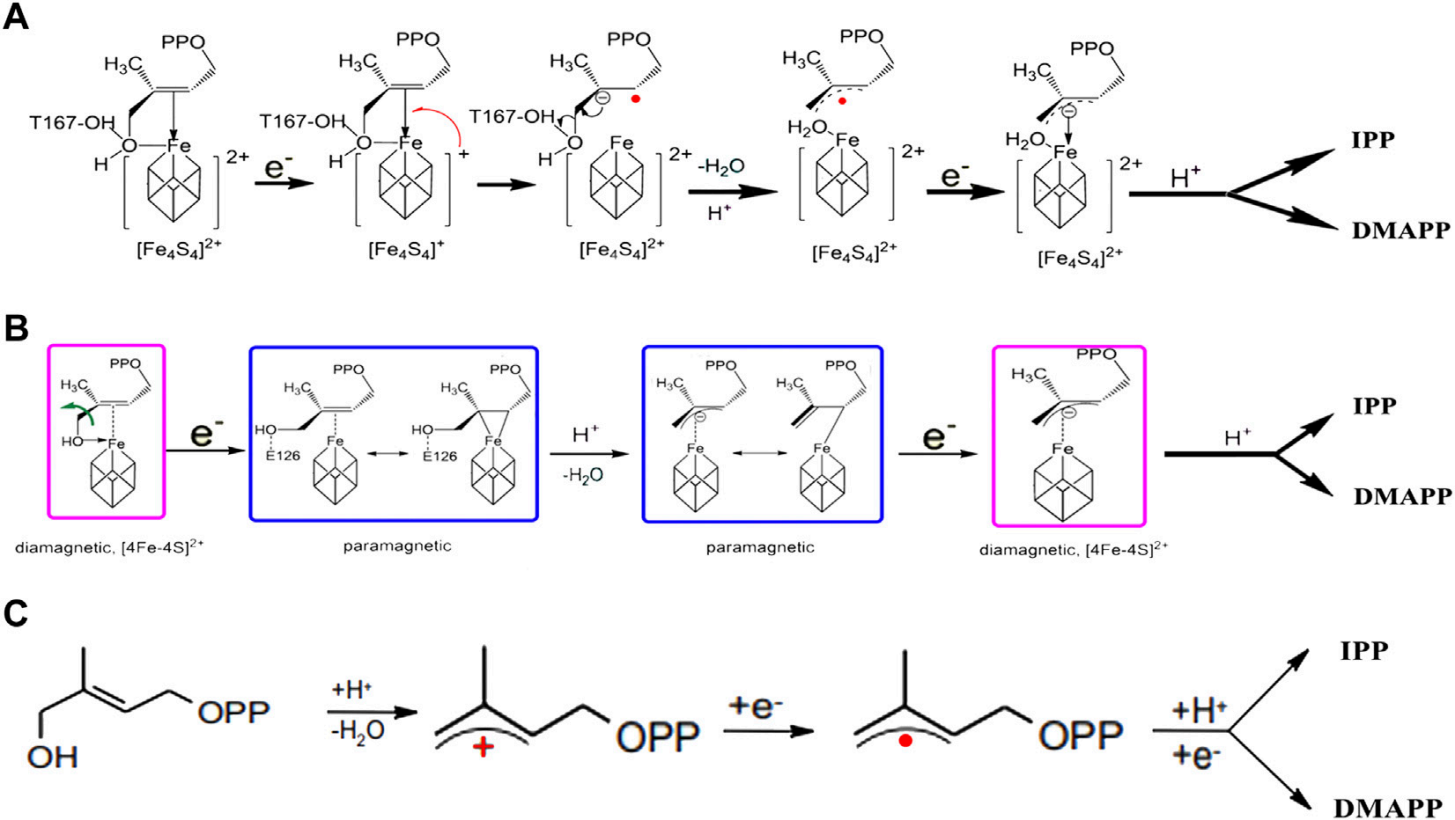
Catalytic reaction mechanism in IspH. **(A)** According to Birch reduction theory; **(B)** according to the Bioorganometallic mechanism; **(C)** according to allyl cation model theory.

The mechanism of enzyme catalysis for IspH is controversial and there have been several proposals related to this (Altincicek et al., 2002; Wolff et al., 2002; Rohdich et al., 2003; Xiao et al., 2008; Xiao and Liu, 2008; Gräwert et al., 2009). Three main hypotheses for the catalytic mechanism have been proposed. In the Birch Reduction Theory (Figure 3A), the process of reduction and dehydroxylation of HMBPP is similar to a Birch reduction reaction. When the HMBPP substrate binds to the protein, an electron is first transferred from the [4Fe-4S] center to HMBPP, which then protonates and loses a H_2_O molecule. After the hydroxyl is lost, HMBPP forms an allyl carbon free radical; the intermediate then accepts a second electron and is protonated to form IPP and DMAPP (Rohdich et al., 2003). The Birch Reduction Theory is regarded as the most likely mechanism occurring for IspH-based catalysis.

The second proposal is the Bioorganometallic Theory (Figure 3B), which was proposed based on the biophysical properties of the complex formed between the HMBPP substrate and the IspH Glu126Ala mutant (Wang et al., 2010). In this mechanism, the substrate hydroxyl group first binds to the IspH [4Fe-4S] cluster and then receives an electron to form an HMBPP-[4Fe-4S] complex, similar to a π or 2-alkenyl/metallacycle complex, which is then dehydrated to form a 1-allyl intermediate. This receives an electron and is reduced to form a 3-allyl intermediate complex, which is finally protonated to form IPP and DMAPP.

The third theory is the allyl cation model (Figure 3C). In this proposal, the metal center, as a Lewis acid, is more conducive to fracture of the C4-OH bond and the subsequent formation of an allyl cation intermediate product, which receives two electrons and is protonated to eventually form the products IPP and DMAPP (Altincicek et al., 2002).

The common point in these three theories is that the initial reaction requires the C4-OH in the HMBPP substrate to be combined with the [4Fe-4S] center in the enzyme, as well as formation of the final reaction product during a prolongation step. It is believed that the proportions of IPP and DMAPP are controlled by the position of the prolongated carbon. Clearly, more studies are required to further delineate the catalytic mechanism of IspH. Additional experiments are also needed to determine how the reaction flux (IPP *vs*. DMAPP) varies in the IspH-catalyzed reaction.

### Catalytic promiscuity of IspH

IspH is the final enzyme in the MEP pathway, and its natural catalytic products IPP and DMAPP are crucial precursors for downstream terpenoids compounds. As the simplest terpenoid, isoprene is an important platform compound that is widely used in the production of rubber. In 1957, Sanadze *et al.* discovered isoprene emission from woody plants (Sharkey and Yeh, 2001). Since then, it has also been found that isoprene is produced by other organisms, including animals, fungi, and bacteria (Kuzma et al., 1995; Sharkey and Yeh, 2001). Isoprene can be synthesized *via* the MEP pathway with DMAPP as the precursor, and this reaction is catalyzed by an isoprene synthase (IspS) that is only found in higher plants (Monson et al., 2013). However, in the study of *Bacillus*, IspH was found to have the same activity for catalyzing the formation of isoprene. Hess *et al.* used transcriptomic analysis to predict the possible genes involved in isoprene production in the MEP pathway of *B. subtilis*, and showed that the overexpression of IspH was negatively correlated with isoprene production (Hess et al., 2013). Julsing *et al.* analyzed all genes in the MEP pathway by means of a conditional knockout strategy, aiming to explore the influence of different genes on the isoprenoids of *B. subtilis* (Julsing et al., 2007)*.* The results showed that the yield of isoprene from the mutant of which the IspH-coding gene *yqfP* was deleted was 9.8 times lower than that in the wild-type strain. Currently, there is no direct evidence that *B. subtilis* IspH is responsible for producing isoprene; however, Ge *et al.* confirmed that IspH derived from an alkaliphilic *Bacillus* sp. N16-5 exhibits isoprene synthase activity (it can catalyze the production of isoprene from endogenous HMBPP and isoamylene from DMAPP) (Ge et al., 2016). To date, IspS has not been found in microorganisms (Köksal et al., 2010). The discovery of an isoprene synthase in microorganisms will undoubtedly be important.

Some studies have reported other novel activities of IspH. IspH from *E. coli* could catalyze the conversion of acetylene to acetaldehyde and ketone through hydration (Span et al., 2012b). Other studies confirmed that IspH can use a number of HMBPP analogs as substrates (Xiao et al., 2011; Wang et al., 2012). Obviously, microbial IspH presents catalytic promiscuity; however, these novel catalytic activities are poorly understood. If the mechanism of the multi-substrate, catalytic function of IspH can be further explored in future studies, it will provide a theoretical basis for engineering IspH, providing new enzyme resources for terpenoid biosynthesis.

### Biotechnological potential

Isoprenoids are a large family, many of which are important industrial compounds with high added values. The simplest is isoprene, which is used as a raw material for the production of rubber. In addition, complicated-structure terpenoids such as limonene, linalool, vitamin E, vitamin K, and β-carotene are antioxidants. Many studies have already reported that isoprenoid compounds can be synthesized in microorganisms *via* either the MEP or MVA pathways (Rohmer, 1999; Kuzuyama, 2002). Using synthetic biology approaches, many studies have achieved high-level production of many isoprenoid compounds in engineered microbes, including in *E. coli*, *Saccharomyces cerevisiae*, and *B. subtilis*. It was proposed that the MEP pathway may present several potential control points, each exhibiting different degrees of control (Li et al., 2018). IspH is a crucial rate-limiting step for amplifying the isoprenoid flux. To increase β-carotene production in *E. coli,* a strong promoter was employed to replace the native promoter IspH in order to increase the isoprenoid flux (Yuan et al., 2006; Suh, 2012). *lytB* (encoding IspH) from *Thermosynechococcus elongatus* was introduced into *E. coli*, resulting in efficient isoprene production (Chotani et al., 2013).

### Development of novel anti-infectives

In addition to serving as a target in MEP pathways to improve the production of terpenes *via* metabolic engineering, IspH can also be used as a target in protein inhibitors for the development of antibacterial drugs. The MEP pathway is absent in mammals but is essential for the survival of many pathogenic bacteria; thus, it provides a new route for the development of novel antibacterial drugs (Rohdich et al., 2005). Most studies have focused on the development of compounds that inhibit the activity of IspH. Reported IspH inhibitors include substrate analogs, pyridine diphosphates, alkyne derivatives, and non-diphosphate compounds.

Two HMBPP analogs were designed, wherein a thiol or amino group replaced the hydroxyl group in HMBPP. A complete kinetic investigation in anaerobic conditions revealed that these analogs were extremely potent inhibitors of *E. coli* IspH, displaying competitive modes of inhibition (Ahrens-Botzong et al., 2011; Janthawornpong et al., 2013). Wang *et al.* studied a set of pyridine derivatives substituted in the ortho, meta, and para positions as potential inhibitors of IspH in *A. aeolicus*, and found that two of them exhibited superior inhibitory potencies (Wang et al., 2010). In addition, Wang *et al.* also demonstrated that alkyne diphosphate can function as an inhibitor of *A. aeolicus* IspH (Wang et al., 2010). O’Dowd *et al.* used an *in silico* approach to screen a series of compounds from ZINC and NCI libraries; the authors identified two drug-like compounds that acted as IspH inhibitors (O’Dowd et al., 2017). However, although a number of potent IspH inhibitors have been discovered, to date, no reports exist on research using these inhibitors against pathogenic bacteria.

## Conclusions and perspectives

The microbial MEP pathway has long been known; however, few studies regarding individual enzymes in the pathway have been reported, particularly the last enzyme in the MEP pathway, IspH. Because IspH contains an iron-sulfur cluster that degrades in air, the technical conditions required for the expression and purification of the protein are relatively strict. However, considering its importance in both the MEP pathway and isoprenoid compound biosynthesis, further research on this protein is required, and should include: 1) Studies for improving the enzyme’s properties, such as stability, catalytic activity, substrate binding, product specificity, and cofactor-binding affinity to further enhance its application potential. 2) Solving the mismatch in the reduction potential issue and the difficulties faced in [4Fe-4S] cluster reduction in IspH. 3) Research on IspH inhibitors has been limited to *in vitro* experiments, therefore future research should focus on *in vivo* experiments to provide a theoretical basis for the development of new antibacterial drugs targeting IspH. A systematic and in-depth study of the biochemical properties and structure–function relationships in IspH will provide valuable information for engineering and using IspH in future biotechnological applications.
